# Collecting Performance Prediction for the Rubber Collector in Horizontal Wellbore Based on AutoML

**DOI:** 10.3390/s25061836

**Published:** 2025-03-15

**Authors:** Shaohua Li, Yang Li, Longlin Chen, Xianbin Wang, Weihang Kong

**Affiliations:** 1 School of Information Science and Engineering, Yanshan University, Qinhuangdao 066004, China; shaunlee0916@163.com; 2 Reservoir Performance Monitoring Center, Shengli Oilfield Company, China Petroleum & Chemical Corporation, Dongying 257000, China; liyang_sinopec@163.com; 3 Logging and Testing Services Company, Daqing Oilfield Company Limited, Daqing 163453, China; dlts_chenll@petrochina.com (L.C.); dlts_wangxianbin@petrochina.com (X.W.); 4 Hebei Key Laboratory of Computer Virtual Technology and System Integration, Qinhuangdao 066004, China

**Keywords:** horizontal well, rubber collector, eccentric prediction of collecting performance status, finite element method, AutoML

## Abstract

To objectively study the influencing factors of rubber collector performance in horizontal wellbores and identify parameter optimization directions, this paper introduces a modeling approach for rubber collectors in horizontal wellbores and develops a corresponding performance prediction method using AutoML(implemented with AutoGluon V1.2.0). Specifically, the study establishes a simulated model of a rubber collector in a horizontal wellbore, simulating its operational state under eccentric conditions due to gravity. It evaluates the impact of rubber elastic element properties (such as material and size) on collecting performance using the integral value of the stress distribution as a metric. Based on the simulated model, an AutoML-based regression model is constructed to predict the collecting performance of the rubber collector under various parameters of the rubber elastic component. The analysis reveals that, as the eccentricity of the rubber collector increases, the most significant influencing factors on collecting performance, in descending order of importance, are axial extrusion range, hardness, radius, bulk modulus, and concave degree. The experimental findings indicate that, during parameter optimization for rubber collectors with varying eccentricities, priority should be given to the aforementioned factors. Additionally, adjusting properties like bulk modulus can enhance the axial extrusion range, thereby effectively boosting the overall collecting performance of the rubber collector in a horizontal wellbore.

## 1. Introduction

Horizontal-well fluid producing profile logging [1,2] serves as the crucial means of mastering the dynamic production process of horizontal wells and ensuring the continuous efficient development of medium- and low-yield fluid horizontal wells [3]. Collecting logging aims to ensure the measuring accuracy of the downhole instrument through improving the flowing velocity of the fluid and decreasing the oil-water surge. For horizontal-well production profile collecting logging technology with oil tubing transporting [4], the collector is connected to the oil tubing in series; when the collector enters the horizontal section of the horizontal well with the tubing, there is a certain gap between the oil tubing and the casing pipe to ensure that the tubing and logging instrument can run smoothly.

However, the oil tubing and in-series logging instruments are in the middle and lower positions of the casing under the influence of gravity when they run into the horizontal measurement section; that is, they are in an eccentric working status. At this time, the packer of the collector may not be in contact with the inner wall of the casing pipe, which makes it difficult for the collector to completely seal the annular space of the oil casing pipe [5] and thus results in leakage loss; the leakage loss could make the collecting logging instrument not realize full collecting testing, thus possibly not meeting the accurate and fine measuring requirements for the production of the different zones in medium- and low-yield fluid horizontal wells. Therefore, conducting research on the interrelationship between the packer of the collector and the contacted casing pipe under different eccentric conditions as well as the influence of the eccentric status on the collecting performance are of great theoretical and practical significance.

In the field of liquid production profile logging in medium- and low-yield field horizontal wells, an electric extruded rubber collector is widely applied due to its high collection efficiency and reliability [6]. In order to ensure that the rubber collector in a horizontal wellbore under eccentric status maintains good collecting performance, combining finite element simulation and the artificial intelligence method to conduct research on the optimizations of the rubber collector and logging sensors has become the focus of the current research. Chen et al. [7] utilize the non-linear finite element method and the axial compression experiment to determine the mechanical constants $C_{10}$ and $C_{01}$ of the model, use the determined parameters to simulate the rubber, and analyze the initial sealing process and intermediate sealing process of the rubber with different hardness values; Wu et al. [8] conclude that the measuring accuracy of the petroleum logging is not only related to the properties of the fluid and the measuring instrument but also closely related to the associated equipment as well as environmental factors, such as the wall thickness of the casing pipe, the outer diameter of the casing pipe, the perforation density, the inner diameter of the wellhead, and so on; they also utilize the Monte Carlo method to simulate the response rules of the porosity to the aforementioned parameters, providing novel insight to improving the logging accuracy in view of assisting the measuring instrument. So far, few studies have been conducted on the interrelationship between the elastic part of the electric extruded rubber collector and the casting pipe as well as the influence rule of the different eccentric degrees on the collecting performance. Automated Machine Learning (AutoML), as a new approach in the field of artificial intelligence, is proposed mainly in response to the fact that traditional machine learning models face many challenges in their applications, especially in feature engineering, model selection, hyper-parameter tuning, and model evaluation. Traditional machine learning applications are time-consuming and error-prone when it comes to feature engineering, model selection is fraught with variables, hyper-parameter tuning is complex and cumbersome, and model evaluation requires a robust validation framework. These steps require significant expertise and time investment, limiting the rapid deployment of models in new environments. These steps require significant expertise and time investment, limiting the rapid deployment of models in new environments. AutoML greatly simplifies the machine learning workflow by automating the steps of feature engineering, model selection, and tuning. In feature engineering, AutoML tools automatically identify and create useful features, reducing the burden on experts. In terms of model selection and tuning, AutoML uses advanced search strategies and model fusion techniques to find the best model architecture and hyper-parameters, reducing manual trial and error. In addition, AutoML models are more versatile and can automatically perform feature engineering and model selection to better adapt to new data. AutoML tools also provide valuable information in feature importance analysis and are therefore widely introduced into engineering applications [9,10]. De Souza et al. [11] employ AutoML technology to predict the diesel oil spectrum and realize a certain improvement compared with the traditional machine learning method; Nikitin et al. [12] establish the prediction model of water detection underground and utilize AutoML technology to conduct parameter-searching optimization on the established model, realizing the prediction of the optimal logging and mining position.

Nevertheless, few studies have reported research about analyzing and optimizing eccentric rubber collectors in horizontal wellbores using AutoML technology. Based on the aforementioned discussions and observations, combining numerical simulation and AutoML technologies and taking into account the interrelationship among the parameters of the rubber elastic part (size, geometry, material properties, frictional coefficient, and axial extrusion range), the contact/equivalent stress generated by the local deformation of the oil casing, and the collecting performance, this paper conducts research on the prediction and optimization of the collecting performance of rubber collectors with different eccentric degrees in horizontal wellbores.

## 2. Simulated Modeling of a Rubber Collector in Horizontal Wellbore

**Figure 1:**
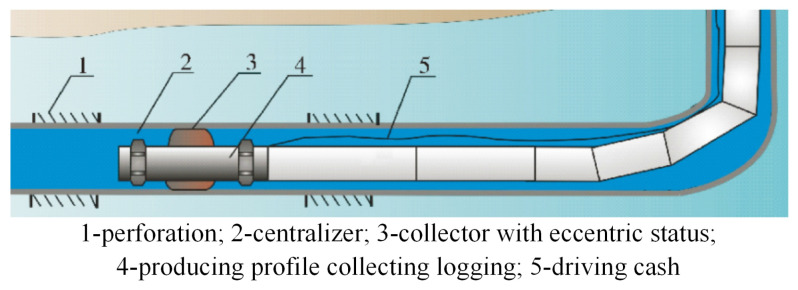
Horizontal−well production profile collecting logging technology with oil tubing transporting.

**Figure 2:**
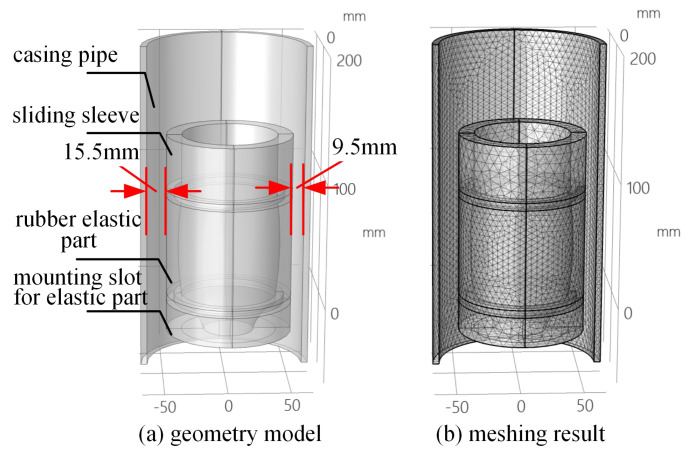
Simplified model and meshing of rubber collector with eccentric status.

Figure 1 represents the horizontal-well production profile collecting logging technology with oil tubing transporting, where the oil tubing and the collecting-type instrument are in series [13,14] to transport. Moreover, setting a centralizer with certain eccentricity ensures that the tubing and instrument can be taken off smoothly. However, due to the effect of gravity on the oil tubing and the instrument in the horizontal wellbore, the collector might become eccentric and thus may not realize measurement for 100% collecting. The rubber elastic part serves as the core component of the electric extruded rubber collector and is thus directly related to the overall performance of the rubber collector. In order to further analyze the overall collecting performance of the rubber collector with different eccentric degrees, the simplified model of the eccentric rubber collector in a horizontal wellbore is established in Figure 2, including the casing pipe, sliding sleeve, rubber elastic part, and mounting slot for the elastic part. For the model in Figure 2, the materials of the casing pipe, sliding sleeve, and mounting slot for the elastic part are ‘structural steel’, characterized by an elastic modulus of 210 GPa, Poisson’s ratio 0.3, density 7850 kg/m^3^, and strength factor 560 Mpa. The material of the elastic part is ‘superelastic rubber’, characterized by an elastic modulus of 0.01 Gpa, Poisson’s ratio 0.5, and density 1100 kg/m^3^. In the field of rubber simulation, the Mooney-Rivlin model is favored for its excellent applicability and convenience. The model can effectively simulate the mechanical behavior of most rubber materials, and, compared with other rubber material models, it has excellent performance in dealing with small and medium deformations, and the process of parameter acquisition is relatively simple, which can usually be realized by basic experimental methods such as a uniaxial tensile test, thus providing convenience for simulation analysis, especially large-scale simulation analysis [15]. Based on the advantages of the Mooney-Rivlin model, it can more accurately simulate the actual working conditions of the rubber collector in a horizontal borehole, especially when it is subjected to compression and deformation. This accuracy helps to improve the reliability of the rubber collector collection performance prediction model, which in turn optimizes its design and parameter configuration. So, the model incorporates the Mooney-Rivlin dual parameter model for enhanced material characterization and analysis. The mounting slot for the elastic part has a ‘fixed constraint’ added. The finite element method usually uses simple functions such as simple primary or quadratic functions to describe the solution in a single cell, so the principle of meshing should follow the physical field in a single cell and cannot change drastically; otherwise, the function cannot describe this drastic change. The accuracy of mesh delineation is crucial as too large a mesh may lead to significant deviations in the simulation results from reality and even non-convergence of the results, while too fine a mesh can improve the accuracy of the simulation, but, at the same time, it also places increased demand on the computer resources and extends the computation time. Taking various factors into consideration, this study adopts the rubber collector meshing results shown in Figure 2b, with a square tetrahedral mesh type: the rubber elastic part uses the ‘corner refinement’ technique, with a total number of 65,116 mesh cells, and the average mesh quality index is 0.8566, which indicates that the mesh quality is high and can meet the demands of simulation and analysis.

Limited by the influence of factors including the inner diameter of the oil casing pipe, axial motor load, and extrusion range, the main parameters of the simplified model of the rubber collector are tabulated in Table 1.

The rubber elastic part serves as the superelastic material, its physical properties are complex, its material and geometry form a double uncertainty, and thus it is difficult to employ an accurate mathematical modeling method to fit its properties. Therefore, the simulated modeling utilizes the Mooney-Rivlin superelastic constitutive model to fit the mechanical properties of the rubber elastic part [16,17], such as the hardness, where the constitutive relation is as follows [18]:

(1) $$\begin{array}{cc} \; & \sigma_{ij}=-pE_{ij}+\\ \; & \frac{2}{\sqrt{I_{3}}}\left[I_{3}\frac{\partial W}{\partial I_{3}}E_{ij}-\frac{\partial W}{\partial I_{2}}B_{ij}^{2}+\left(\frac{\partial W}{\partial I_{1}}+I_{1}\frac{\partial W}{\partial I_{2}}\right)B_{ij}\right] \end{array}$$

where $\sigma_{ij}$ is the component of Euler stress tensor, *p* is the hydrostatic pressure, $B_{ij}$ is the component of the left Cauchy-Green deformation tensor, and $I_{1}$, $I_{2}$, and $I_{3}$ are the first component, the second component, and the third component of the deformation tensor.

Taking the axial direction as the main strain direction and considering that the volume of rubber under uniaxial loading is approximately incompressible, three main principal drawing ratios are set as $\lambda_{1}=\lambda$ and $\lambda_{2}=\lambda_{3}=1/\sqrt{\lambda }$, where λ is the variable, $\lambda_{1}\lambda_{2}\lambda_{3}=1$, and the total bulk volume does not change. Thus, the left Cauchy-Green deformation tensor B could be obtained as

(2) $$B=F\cdot F^{T}={\left[\begin{array}{ccc} \lambda & 0 & 0\\ 0 & \frac{1}{\lambda } & 0\\ 0 & 0 & \frac{1}{\lambda } \end{array}\right]}^{2}=\left[\begin{array}{ccc} \lambda^{2} & 0 & 0\\ 0 & \frac{1}{\lambda } & 0\\ 0 & 0 & \frac{1}{\lambda } \end{array}\right]$$

The three invariants of the deformation tensor can be obtained from F as

(3) $$I_{1}=\mathrm{tr}B=\lambda^{2}+2\lambda^{-1}$$

(4) $$I_{2}=\left[I_{1}^{2}-\mathrm{tr}B^{2}\right]/2=\lambda^{-2}+2\lambda$$

(5) $$I_{3}=1$$

In the engineering calculation of rubber materials, the Mooney-Rivlin strain-energy function is widely applied and the elastic strain energy density function $W\left(I_{1},I_{2}\right)$ could be expressed as

(6) $$W\left(I_{1},I_{2}\right)=C_{10}\left(I_{1}-3\right)+C_{01}\left(I_{2}-3\right)$$

where $I_{1}$ and $I_{2}$ represent the equal-body invariant, $C_{10}$ and $C_{01}$ represent the Mooney-Rivlin parameters, and W represents the elastic strain energy.

The mechanical deformation method of the rubber elastic part is uniaxial compression, and the first Piola-Kirchhoff stress of the uniaxial deformation could be expressed as

(7) $$P_{1}=2\left(1-\lambda^{-3}\right)\left(\lambda C_{10}+C_{01}\right)$$

where λ represents the main expansion coefficient of the uniaxial compression and $P_{1}$ represents the first Piola-Kirchhoff stress of the uniaxial deformation.

The relation between the hardness of the rubber and the Mooney-Rivlin parameter [17] is reported in Table 2.

## 3. Simulated Analyses of the Collecting Performance of the Rubber Collector with Different Eccentric Degrees

**Figure 3:**
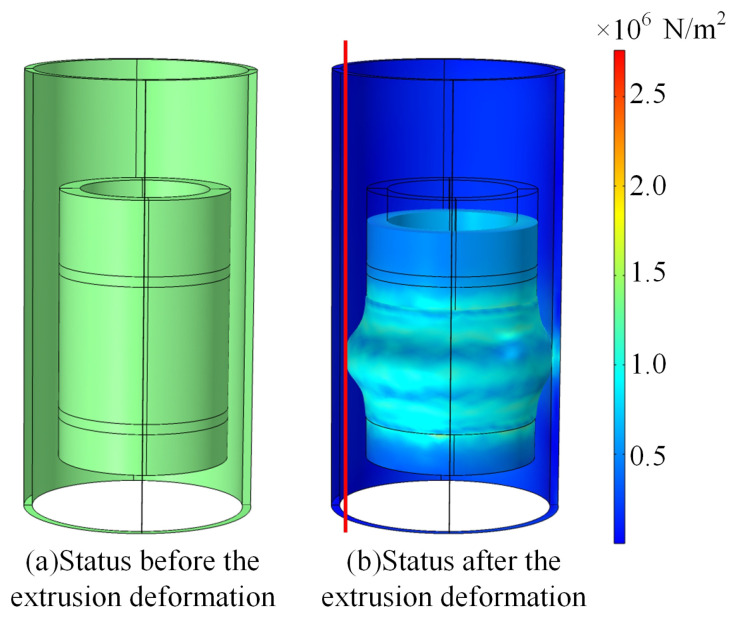
Status of the rubber collector with eccentricity of 3 mm before and after extrusion deformation.

Figure 3 shows the status of the rubber collector with the eccentricity of 3 mm before and after the extrusion deformation. When the rubber collector assumes the status of the extrusion deformation from the initial status, the main difference between the eccentric status and the coaxial status is that the deflected side of the collector would be in contact with the oil casing in advance and generate contact stress with the casing, while the deflected negative direction (red line on the left of the Figure 3) has not been in contact with the oil casing. At the time of the eccentric status, the axial extrusion depth is 18 mm; when the extrusion depth becomes larger, the deviated side of the elastic part gradually contacts with the oil casing, resulting in less contact stress than the deflected side, which is the weakest part of contacting in the annular space of the oil casing. Then, the integral value $K_{L}$ of the contact stress at the weakest part is utilized to evaluate the collecting performance of the collector model [18], where the expression of $K_{L}$ is

(8) $$K_{L}=\int V_{L}dL$$

where $V_{L}$ represents the distribution of stress in the casing pipe and *L* is the length of the casing pipe.

The parameters of the rubber collector with the eccentricity of 1–3 mm are shown in Table 3, and, through traversal, the convergence models of the rubber collector with the eccentricity of 1–3 mm are obtained.

**Figure 4:**
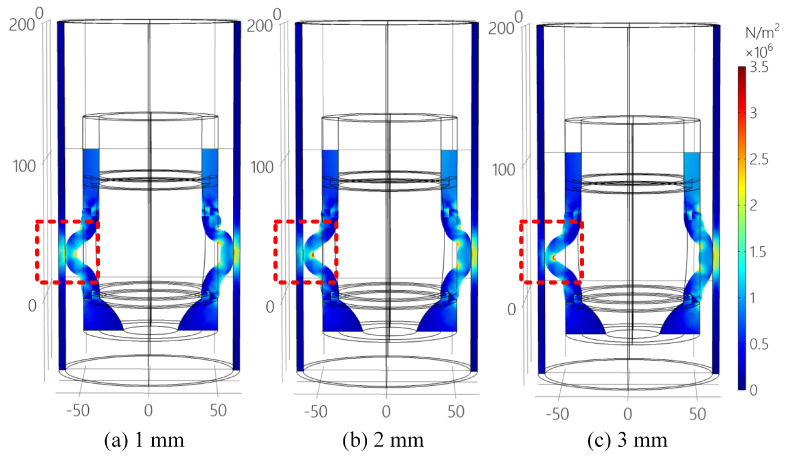
XZ Please let us know if further adjustments are required. plane slices of the rubber collector with eccentricity of 1–3 mm.

Figure 4 displays the XZ plane slices of the rubber collector with the eccentricity of 1–3 mm. It could be observed from Figure 4 that, under the eccentric status, the distribution of stress of the rubber elastic part is not uniform: the eccentricity results in a larger contact area between the deflected side of the collector and oil casing, resulting in larger contact stress, while the deviated side has less stress. Moreover, the greater the degree of eccentricity, the larger the contact area between the collector and the oil casing, the greater the stress on the deflected side, and the greater the stress on the deflected side of the collector with large internal deformation. Based on this, the side of smallest set-down loading, that is, the largest deviated side, is taken as the discriminant interface and the evaluation criterion KL is used as the evaluation method for the performance of the collector.

**Figure 5:**
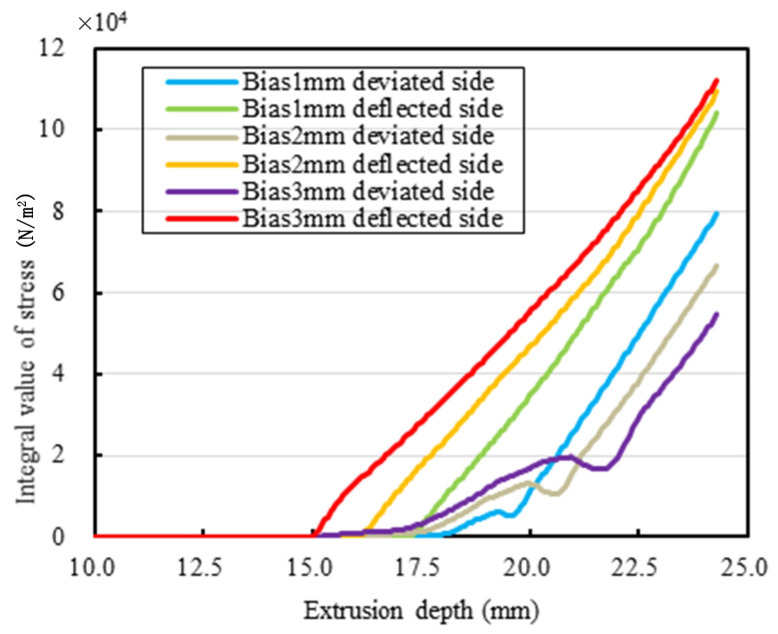
Curve of the integral value of stress changing with extrusion depth when the eccentricity is 1–3 mm.

Figure 5 shows the curve of the integral value of stress changing with the extrusion depth when the eccentricity is 1–3 mm. The following results could be analyzed from Figure 5: with the increase in the extrusion depth, the rubber collector gradually approaches and seals the casing pipe, and deflected side of the rubber collector is first in contact with the casing pipe; under the same extrusion depth, the larger the eccentricity, the larger the stress integral value of the deflected side, and, when the eccentricity is 3 mm, the stress integral value is the largest; the deviated side needs a larger extrusion depth to contact the casing pipe, and, the larger the eccentricity, the smaller the stress integral value is, the fluid is more likely to pass through the region, and the collection performance of the rubber collector shows a decreasing trend. When the eccentricity is 3 mm, the smaller the stress integral, the weaker the rubber collector performance. With the increase in the eccentricity, the integral value of the stress $K_{L}$ of the deviated side gradually decreases and thus the collecting performance is tending downward. In particular, at an eccentricity of 5 mm, the stress between the deflected side of the rubber collector and the casing pipe increases significantly, while the stress on the other side decreases significantly, leading to easier fluid leakage from the region of lower contact stress. In addition, as the eccentricity increases, the extrusion depth of the rubber collector needs to be larger to achieve an effective seal, which further increases the deformation difficulty of the rubber collector and leads to a decrease in collection performance. We could summarize the following conclusions: when the eccentricity increases, the deflected side is first in contact with the casing pipe, and it is difficult for the deviated side to contact the casing pipe, seriously affecting the collecting performance of rubber collector. Due to the fact that the volume of the superelastic material is almost constant under stress deformation, the shoulder protrusion would be generated and thus yields the hindering effect after the extrusion deformation of the rubber elastic part. When the inward extrusion continues, the solver would not converge, which is due to the fact that the finite deformation of rubber exceeds the “deformation limit” and does not accord to the Mooney-Rivlin constitutive model. During the practical engineering applications, exceeding the “elastic limit” would lead to the reduction in durability of the rubber elastic part and even permanent damage.

In order to study the simulated experiment with larger eccentricity, the effect of the bulk modulus on the collecting performance is considered. The bulk modulus describes the ratio of volume change of hyperelastic materials such as rubber due to volume load. The volume with the intensity of pressure *P* is $V_{0}$; if the change in the intensity of pressure is dP and the change in the volume is dV, then there is

(9) $$K=\frac{dP}{dV/V_{0}}$$

where *K* is the bulk modulus.

The relation among the bulk modulus, the elasticity modulus (Young’s modulus), and the Poisson ratio could be expressed as

(10) $$K=\frac{E}{3\left(1-2\mu \right)}$$

where *E* is elasticity modulus (Young’s modulus) and μ is the Poisson ratio.

**Figure 6:**
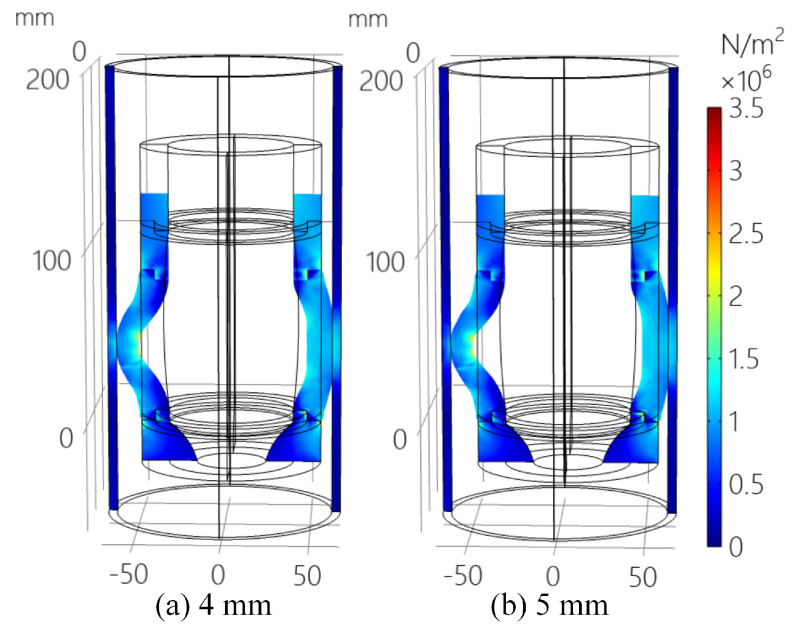
XZ plane slice diagram of the simulation results with eccentricity of 4–5 mm.

**Figure 7:**
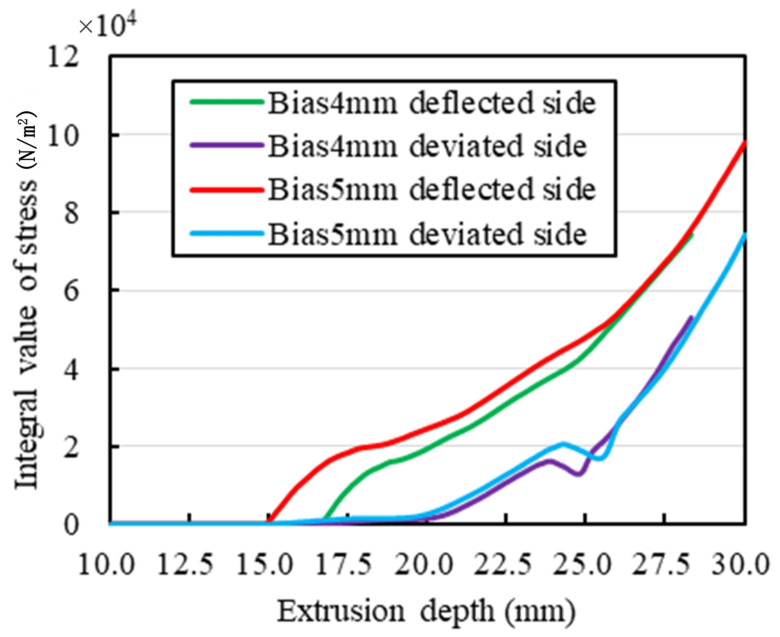
Relation curve between extrusion depth and the flow performance of the rubber collector.

Substituting the parameters of the 58 HA superelastic rubber *E* = 4.268 MPa and μ = 0.46, then *K* = 17.783 MPa and is used as the initial experimental value. After the more detailed large-scale parameter traversal, removing the non-convergent solution, based on the parameters in Table 4, the XZ plane slice diagram of the simulation results with the eccentricity of 4–5 mm are obtained, and the relation curve between extrusion depth and the flow performance of the rubber collector is also obtained, as shown in Figure 6 and Figure 7.

Figure 6 shows the XZ plane slice diagram of the simulation results with the eccentricity of 4–5 mm. It could be observed from Figure 6 that, under the eccentric condition, the stress distribution of rubber elastic part is extremely uneven, with the deflected side of the collector having a larger contact area with the casing and also generating more stress, while the deviated side generates less stress. Figure 7 shows the relation curve of the extrusion depth and the collecting performance of the rubber collector with the eccentricity of 4–5 mm. When the eccentricity is 5 mm, the extrusion depth could be up to 30 mm; while the eccentricity is 4 mm, the extrusion depth is at most 28 mm. The reason for the above phenomenon might be that, at the deflected side, the rubber elastic part might generate deformation due to the advanced contact and simultaneously the deviated side could be in contact with the casing pipe more easily. When the extrusion depth is less than 25.5 mm, the larger the degree of eccentricity, the larger the stress integral value of the deflected side and, on the contrary, the smaller the stress integral value of the deviated side; when the extrusion depth is larger than 25.5 mm, the stress integral values of the deflected side and the deviated side with different degrees of eccentricity do not have much influence, which is due to the fact that the rubber collector contact casing is basically the same for different eccentricity levels. However, in order to realize a larger value of the extrusion depth, the integral value of the distribution of stress of the model with the eccentricity of 5 mm would be larger than that with the eccentricity of 4 mm.

## 4. Prediction of the Collecting Performance of the Rubber Collector in Horizontal Wellbore Based on AutoML

In order to solve the non-convergence problem of the model caused by a large number of parameter traversals, the prediction model of the collecting performance of the rubber collector with different eccentric degrees is established using AutoML technology. The AutoML technology [19] simplifies the parameter traversal process through the end-to-end automatic process and plenty of model fusions, and the fitting model could also be determined automatically with the aid of the searching strategy of the AutoML technology, avoiding significant exploration due to domain adaptation.

### 4.1. Dataset Collection

The collecting performance of the rubber collector is related to multiple features, and, based on the simulation results of the rubber collector with different eccentric degrees, the dataset is established, whose statistical information is shown in Table 5.

The dataset used in Table 4 is based on the COMSOL(v6.1) simulation model of different pressure columns extruding a rubber collector under different eccentricity degrees to calculate the stress integral value (K_l) of the collector deviation side in each group as the prediction label. The rubber parameters of the feature set include the eccentric degree (bias), the axial extrusion range (press), the Mooney-Rivlin dual parameters ($C_{10}$ and $C_{01}$), the inner diameter of the rubber (Rubber_r), the length of the rubber (Rubber_height), the thickness of the rubber (Rubber_thick), the bulk modulus (*K*), and the thickness of the concave rubber. After de-weighting and missing value processing of the null values existing in the data due to duplicated data among the exported data and non-converged results, the specific parameter information of the dataset of rubber collector in eccentric state is obtained as shown in Table 4.

### 4.2. Basic Architecture of the Prediction Model of the Collecting Performance Based on AutoML

**Figure 8:**
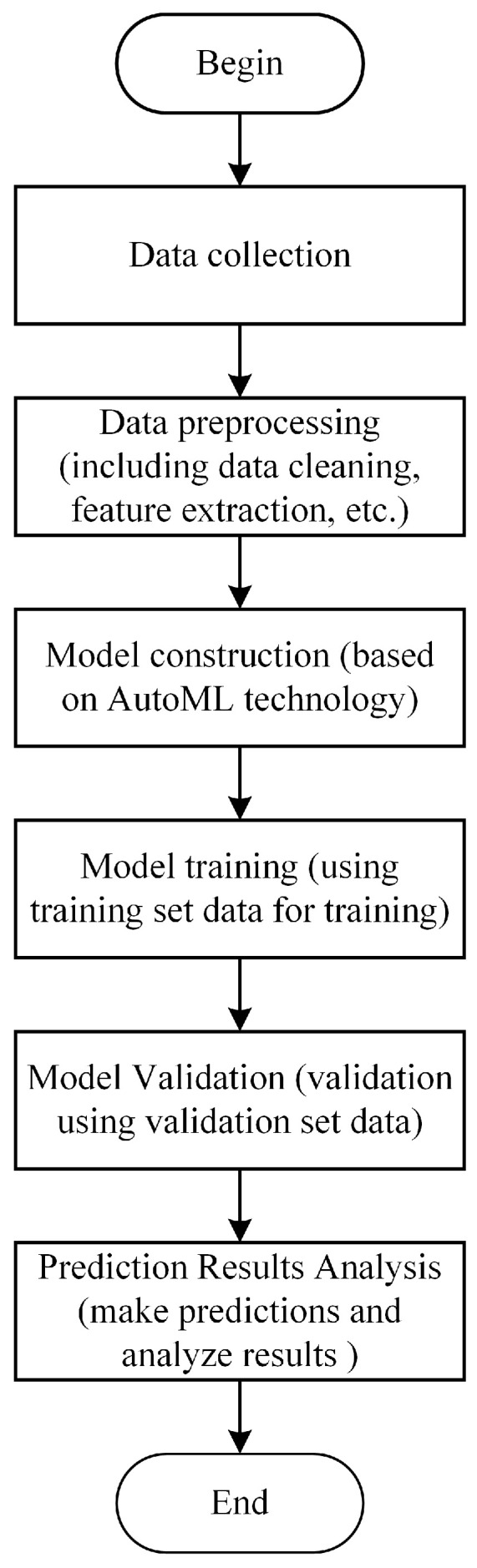
Flow chart of collecting performance prediction method for rubber collector based on AutoML.

**Figure 9:**
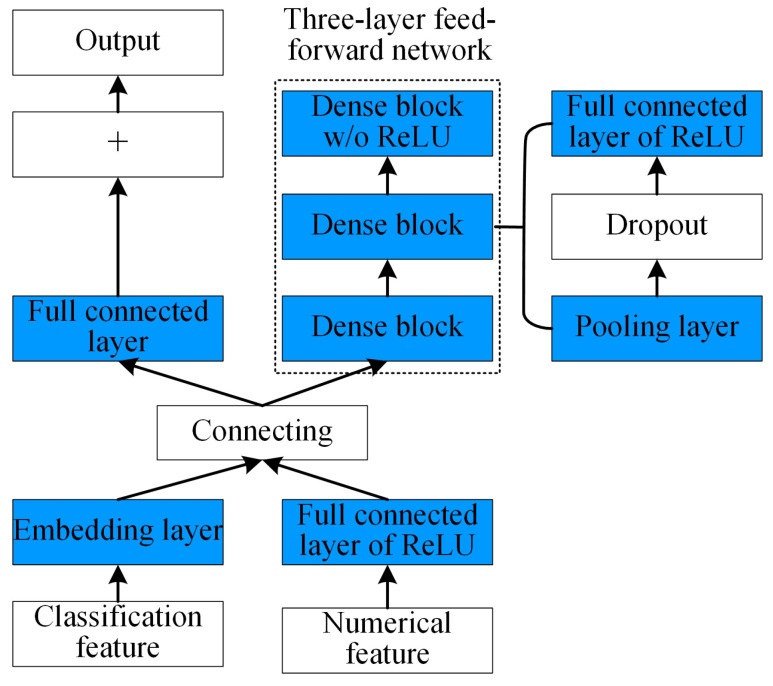
AutoGluon neural network structure.

The AutoGluon automatic optimization machine learning model based on AutoML includes data pre-processing, model architecture searching, hyper-parameter optimization, and so on, as shown in Figure 8. The data pre-processing contains the classification and the formatting for the inputting sampling data; the model architecture searching involves the automatic architecture engineering and makes the machine learning model find the optimal design. The hyper-parameter optimization involves the searching and selecting of the optimal structure of the machine learning model [20]. The basic architecture of the multi-layer stacking model for the collecting performance prediction fusing multiple models is shown in Figure 9. For the architecture in Figure 9, the first layer contains multiple basic models, whose outputs are connected, and the connected outputs are transported into the next layer composed of multiple stacking models. The stacking models serve as the basic models of the additional layer.

### 4.3. Model Fusing with the Machine Learning Algorithm

AutoGluon consists of a series of custom models, including the neural network algorithm, the random forest algorithm, the extremely randomized tree algorithm, two boosting tree algorithms (CatBoost and LightGBM), and the ensemble learning algorithm.

**(1) Neural network algorithm**

The neural network model is established utilizing MXNet. Considering that the collected dataset is composed of multiple types of data, the feed-forward network is the preferred framework for constructing a neural network model. AutoGluon is the first AutoML toolkit to use an embedded layer, which uses linear short connections to connect directly to the network’s output prediction. Each dense block module in the neural network consists of several convolutional layers, pooling layers, ReLU activation function layers, batch normalization layers, and random deactivation regularization (dropout). In this paper, the Adam optimization function with penalty reduction, early stopping, and the L1 loss function are used to train the neural network model, and the optimized network structure is shown in Figure 9.

Different from the pure feed-forward network architecture, AutoGluon-Tabular introduces an embedding layer for each classification feature that matches a learnable component for each classification feature before it is used by subsequent feed-forward layers, and the embedding dimension is selected proportionally according to the number of unique categories in the feature. The embedding layer of the classification feature is then connected with the numerical feature into a large vector that feeds both into a three-layer feed-forward network and is directly connected to the output prediction through a linear jump (similar to the residual network family).

**(2) Random forest algorithm**

Random forest algorithm is a kind of algorithm integrating multiple weak classifiers, whose key is to select the optimal classification and regression based on the discriminant results of multiple random decision trees. Decision tree is the core of random forest, and the optimal generation of decision tree is guaranteed according to information gain. The information entropy $\left(D\right)$ of dataset *D* is calculated according to its basic principle.

(11) $$H\left(D\right)=-\sum_{i=1}^{n}p_{i}log_{2}p_{i}$$

where $p_{i}$ represents the probability of the *i*-th sample.

The information entropy of the dataset $D\;\left(x_{1},x_{2},x_{3}\cdots x_{n}\right)$ is calculated, as well as the conditional entropy between each feature $x_{i}$ and dataset *D*. Information entropy can be used as a measure of uncertainty of dataset *D*. The conditional entropy $H\left(D|X\right)$ is entropy change caused by the variable $x_{i}$ under the generation of dataset *D*.

The conditional entropy of *D* of *X* under given conditions is expressed as

(12) $$H\left(D|X\right)=-\sum_{i=1}^{n}p_{i}H\left(D|X=x_{i}\right)$$

The information gain of the feature *A* on the training dataset *D* is expressed as

(13) $$g\left(D,A\right)=H\left(D\right)-H\left(D|A\right)$$

The advantage of the random forest algorithm is that it can select features. The larger the information gain rate of its features, the stronger the ability of the features to reduce the entropy value, and the stronger the ability to reduce the data uncertainty.

**(3) LightGBM algorithm**

In machine learning, GBDT (gradient boosting decision tree) algorithm based on gradient lifting tree is one of the algorithms recognized in the industry with a better fitting effect for real distribution [12]. It adopts addition model and iterates continuously to reduce the error residual value of the previous epoch so as to achieve the effect of data classification or regression. This model has the advantages of good training effect and flexible processing of all kinds of data. The GBDT algorithm process for binary classification is as follows:

Firstly, the prior information is used to initialize the learner $F_{0}\left(x\right)$, where $p\left(y=1|x\right)$ is the ratio of y = 1 in the training sample.

(14) $$F_{0}\left(x\right)=lg\frac{p\left(y=1|x\right)}{1-p\left(y=1|x\right)}$$

Then, *M* classification regression trees are established m=1,2,⋯,M. For i=1,2,⋯,N, the residual value $r_{m,i}$ corresponding to the *m*-th tree is calculated as the following:

(15) $$r_{m,i}=-{\left[\frac{\partial L\left(y_{i},F\left(x_{i}\right)\right)}{\partial F\left(x\right)}\right]}_{F\left(x\right)=F_{m-1}\left(x\right)}=y_{i}-\frac{1}{1+e^{-F\left(x_{i}\right)}}$$

For n=1,2,⋯,N, the *m*-th regression tree is calculated by using the fitting data of CART regression tree $\left(x_{i},r_{m,i}\right)$, and its corresponding leaf node region is $R_{m,j}$, where $j=1,2,\cdots ,J_{m}$, and $J_{m}$ is the number of the leaf nodes of the *m*-th regression tree. The best fitting value is calculated for each leaf node region, and the strong learner is updated as follows:

(16) $$C_{m,j}=\frac{\sum_{x_{i}\in R_{m,j}}r_{m,j}}{\sum_{x_{i}\in R_{m,j}}\left(y_{i}-r_{m,i}\right)\left(1-y_{i}+r_{m,i}\right)}$$

(17) $$F_{m}\left(x\right)=F_{m-1}\left(x\right)+\sum_{j=1}^{J_{m}}C_{m,j}I,\left(x\in R_{m,j}\right)$$

The final strong learner $F_{M}\left(x\right)$ is integrated and could be expressed as

(18) $$F_{M}\left(x\right)=F_{0}\left(x\right)+\sum_{m=1}^{M}\sum_{j=1}^{J_{m}}C_{m,j}I,\left(x\in R_{m,j}\right)$$

LightGBM (Light Gradient Boosting Machine) is an improved GBDT algorithm framework model proposed on the basis of GBDT algorithm [13], which adopts the histogram-based segmentation algorithm instead of the traditional pre-sorting traversal algorithm. Compared with GBDT algorithm, the proposed improved GBDT algorithm has the advantages of faster parallel training efficiency, lower memory consumption, higher accuracy, increased suitability to deal with massive data, and effectively preventing over-fitting.

**(4) Ensemble learning algorithm**

**Figure 10:**
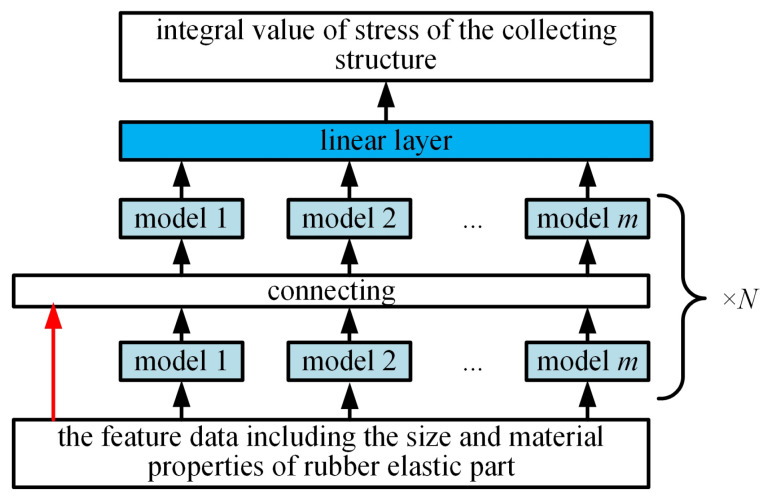
Multi-layer stacking model for the prediction of the collecting performance.

AutoGluon uses a multi-layer stacking strategy to improve the accuracy of the prediction, combining the prediction sets from multiple models to execute a single model and thus greatly reducing the variance of the final prediction. To avoid algorithm selection and hyper-parameter optimization, AutoGluon simply reuses all the basic-layer models as stacks. These models are machine learning models of any type and have the same hyper-parameter values. The predictions of the underlying model are connected in series with the original data features as input vectors for the stacked model. As a result, a higher stack could revisit the original data characteristics. In the last layer of the stacking model, the set selection method is used to gather the predicted results of the stacking model in a weighted way. By repeating the aforementioned process, the multi-layer stacking model is obtained. Figure 10 shows the multi-layer stacking strategy and the use of two-layer stacking of AutoGluon.

### 4.4. Training Process

Step 1: Split the training set and the testing set. The 4789 samples in dataset are split into 3591 samples (75%) for training and 1198 samples (25%) for testing. The validation set utilizes the k-fold method to split.

Step 2: In the hyper-parameter tuning process, we employ k-fold cross-validation and bagging integration strategies to fully utilize the dataset. Specifically, we divided the dataset into 5 parts and used 4 of them sequentially for training during the training process and the remaining 1 part for validation. This process ensures that the entire dataset is used for validation and that each sub-model is adequately trained and validated. For each sub-model, we adjust the hyper-parameters based on its performance on the validation set to optimize the predictive performance of the model. In this process, the hyper-parameters we mainly focus on include the number of layers, convolutional kernel size, learning rate, etc., of the neural network; the number of trees, maximum depth, etc., of the random forest; and the learning rate, iteration number, and number of leaf nodes, etc., of LightGBM. The final model architecture was set as a 4-layer stacked structure with a maximum size of 335, and the specific parameter configurations are shown in Algorithm 1.

Step 3: Model training. The split training set is utilized to train the collecting performance prediction model, and the detailed training process is the execution of Algorithm 1.

Step 4: Test the model. The split testing set is tested on the multiple trained models and the combined model, and the error between the predicted results and the real values of the models is calculated to evaluate each model. For the prediction models, the RMSE, MAE, and R^2^ are usually taken as the evaluating metrics. Since the integral value of the stress of the rubber collector has a larger order of magnitude, the normalized NRMSE and NMAE are adopted, whose calculation formula is as follows:

(19) $$\mathrm{RMSE}=\sqrt{\frac{\sum_{i=1}^{n}{\left({\hat{y}}_{t}-y_{t}\right)}^{2}}{n}}$$

(20) $$\mathrm{NRMSE}=\frac{\mathrm{RMSE}}{y_{max}-y_{min}}$$

(21) $$\mathrm{MAE}=\frac{\sum_{t=1}^{n}\left|{\hat{y}}_{t}-y^{t}\right|}{n}$$

(22) $$\mathrm{NMAE}=\frac{\mathrm{MAE}}{y_{max}-y_{min}}$$

where $y_{t}$, ${\hat{y}}_{t}$, $y_{max}$, and $y_{min}$ are the real value, the predicting value, the maximum real value, and the minimum real value.
**Algorithm 1:** Detailed training process of the collecting performance prediction. Training strategy of the prediction model of the collecting performance of the eccentric rubber collector based on AutoGluon**Input:** Data (*X*: feature of rubber elastic part, *Y*: collecting performance); *M*: set of sub-models; *L*: stacking layer number.**Output:** Prediction result of collecting performance of each model.1:automatic feature engineering2:**for** *l* = 1 to *L* do integrating each layer, *L* = 43: **for** *i* = 1 **to** *n* **do** *n*-times repetition, *n* = 24:  Divide the data into *k* parts randomly ${\left\{X_{j},Y_{j}\right\}}_{j=1}^{k}$, *k* = 55:  **for** *j* = 1 **to** *k* **do** *k*-fold cross-validation and bagging integration6:   **for** each model type *m* in *M* **do**7:     train the model *m* on $X^{-j}$, $Y^{-j}$8:    validate on $X^{j}$, and the prediction value ${\hat{Y}}_{m,i}^{j}$ of each fold9:    
**end for**
10:   
**end for**
11:  
**end for**
12:Average over the ${\hat{Y}}_{m,i}^{j}$ of all folds, ${\hat{Y}}_{m}={\left\{\frac{1}{n}\sum_{i}{\hat{Y}}_{m,i}^{j}\right\}}_{j=1}^{k}$13:Update *X*, *X* = concatenate (*X*, ${\left\{{\hat{Y}}_{m}\right\}}_{m\in M}$)14:**end for**

### 4.5. Prediction Result Analysis

**Figure 11:**
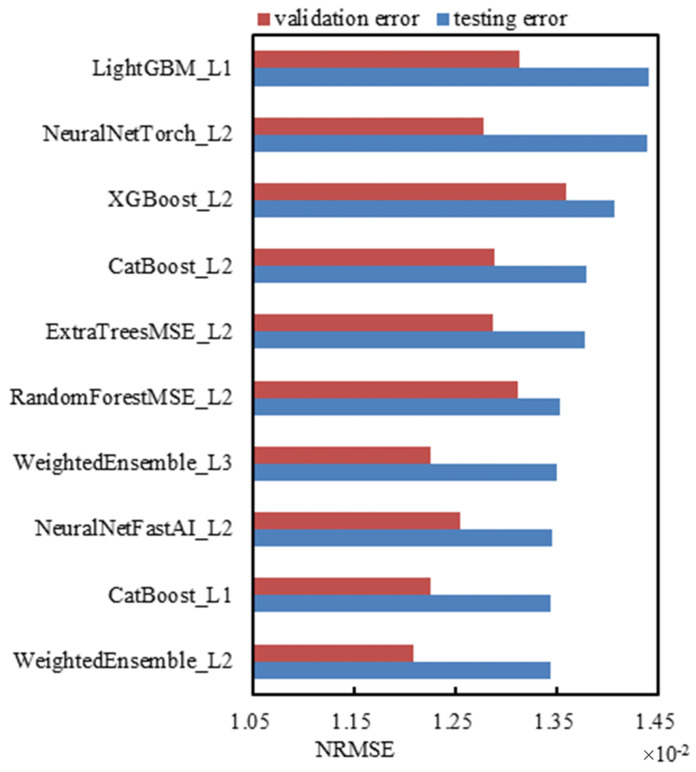
Testing error and validation error of each model layer.

Based on the aforementioned prediction models, the innovation of this study is the first application of the AutoML technique to predict the performance of the rubber collector under eccentricity. Compared with the traditional methods, AutoML significantly improves the prediction accuracy and efficiency through automated feature engineering and model selection. In particular, the use of multi-layer stacked models enables the model to automatically fuse the prediction results of multiple sub-models, further improving the robustness of the prediction. A four-layer stacking model is trained on the datasets of different eccentric degrees and the 21 independent sub-models (including different-layer stacking sub-models). The top 10 models with the best testing results are selected, and the drawn bar diagrams of the testing error and validation error of the models in each layer are shown in Figure 11.

The vertical axis in Figure 11 represents each model, where WeightedEnsemble indicates the output results of the ‘linear layers’ after multiple models are stacked, and the number of Ln after the ‘_’ represents the layer number of the stacking integration. The horizontal axis represents the normalized root mean square error NRMSE. According to the definition, the model with smaller error is better than the predicted result. The validation error is also added in Figure 11 for comparison.The analysis in Figure 11 shows that the model with the best results is “WeightedEnsemble_L2”; that is, the weighted output of the second stacking layer has the lowest errors in both the testing set and the verification set, proving the validity of the multi-layer stacked network. But, it is not the case that the higher the stacked layers the better the prediction. For example, the testing error and validation error of “WeightedEnsemble_L3” are higher than those of “WeightedEnsemble_L2”. There is no four-layer stacking model among the top 10 best-performing models, suggesting that increasing the size of the model indefinitely does not lead to a better result.

**Figure 12:**
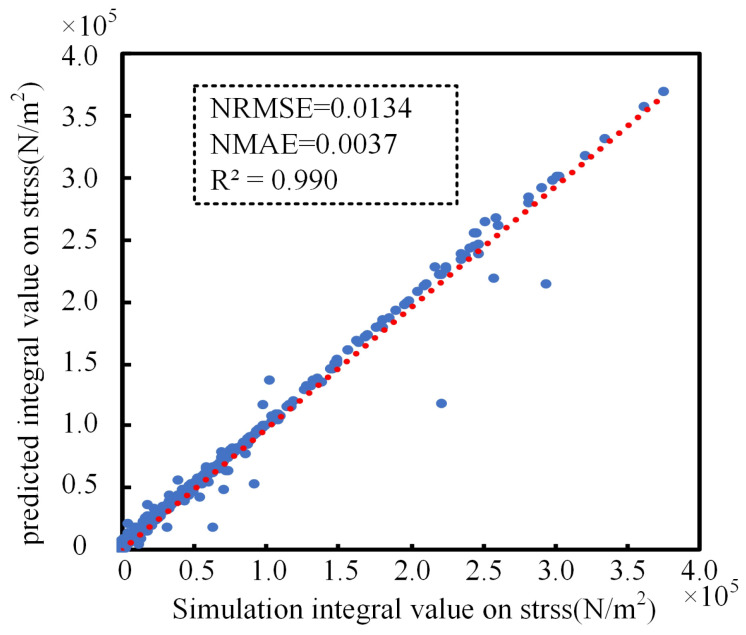
Prediction results on testing.

The optimal “WeightedEnsemble_L2” is adopted for prediction, whose NRMSE on the validation set is 0.0121 and NRMSE on the testing set is 0.0134. The prediction results of the model on the testing set are shown in Figure 12. From Figure 12, it can be seen that the prediction results can fit the simulation results well, and the goodness of fit R2 reaches 0.990, which indicates that the model has captured the relationship between the size, hardness, and other material properties of the rubber and the collector flow performance, and the fitting is better, and it can be used for the prediction of the rubber collector’s flow performance under the eccentricity working condition.

**Figure 13:**
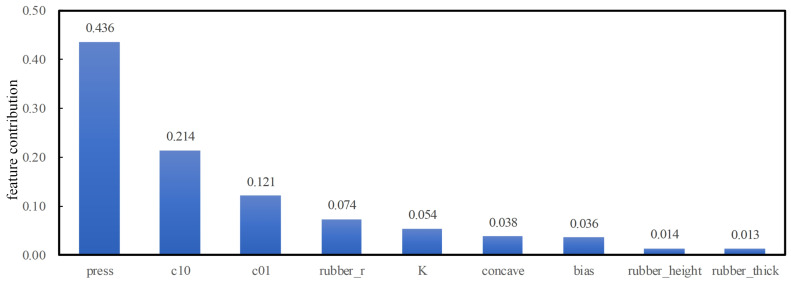
Correlation of integral value of stress with each feature.

The feature importance function is used to output the correlation between features and integral values of stress (i.e., feature contributions), as shown in Figure 13. As can be analyzed from Figure 13 in terms of feature importance, the features such as extrusion depth, hardness of elastic part of rubber, inner diameter and bulk modulus have the most significant effect on the collection performance of rubber collectors. Among them, the collection performance of rubber collectors with different eccentricity is most affected by the extrusion depth, which is consistent with the fact that collectors need to be fully expanded for collection in practical applications. In addition, the hardness, inner diameter, and bulk modulus of rubber elastic part also have a great influence on the collecting performance, and these characteristics are important for guiding significance in the optimization of the collector, which can provide us with strong support for optimizing the design and parameter configuration of the rubber collector.

## 5. Conclusions

(1) An increase in eccentricity has a significant negative effect on the collection performance of the rubber collector, leading to a significant decrease. Through simulation analysis, it was found that the performance loss caused by eccentricity can be effectively compensated by increasing the extrusion depth and adjusting the bulk modulus of the rubber.

(2) Using AutoML technology, the model construction and prediction of the collection performance of rubber collectors under different eccentricity conditions were carried out. A multi-layer stacking structure was adopted, which effectively avoids the difficulties of model optimization and parameter adjustment caused by domain adaptation, and it demonstrated less error and a higher fitting degree compared with a single-layer model.

(3) The results of the predictive analysis show that the size and material properties of the elastic part of the rubber have a significant effect on the collection performance of the rubber collector under different eccentricities, with extrusion depth being the key factor affecting performance. In order to optimize the rubber collector performance, key parameters such as hardness, inner diameter, and rubber bulk modulus should be prioritized, and the range of extrusion depth should be enlarged by adjusting the bulk modulus, which will in turn enhance the collection performance.

(4) Despite the progress achieved in this study, actual data are lacking on the rubber collector performance in extreme environments, such as high temperature and high pressure, which limits the generalization ability of the model under extreme conditions. Therefore, further validation is urgently needed. Future research will focus on exploring more complex material models (e.g., viscoelastic and Mullins effect models) to more accurately simulate the mechanical behavior of rubber materials under real-world operating conditions. In addition, we will expand the dataset, especially by adding experimental data under extreme environmental conditions, to improve the generalization ability of the model.

## Figures and Tables

**Table 1:** Main parameters of the simplified rubber collector model.

Name	Value	Note
Pipe_thick	5 [mm]	Thickness of casing pipe
Pipe_height	200 [mm]	Length of casing pipe
Pipe_r	62.5 [mm]	Inner diameter of casing pipe
Rubber_r	32–38 [mm]	Outer diameter of rubber
Rubber_thick	9–15 [mm]	Thickness of rubber
Rubber_height	80–120 [mm]	Length of the rubber
Press	0–35 [mm]	Axial extrusion range
Concave	8–14 [mm]	Thickness of concave center
*K*	3.56–21.34 [MPa]	Bulk modulus of elastic part
Bias	1–5 [mm]	Eccentric degree

**Table 2:** Parameters of the rubber collector with eccentricity of 1–3 mm.

Name	Value	Note
Rubber_r	38 [mm]	Outer diameter of the rubber
Rubber_thick	12 [mm]	Thickness of the rubber
Rubber_height	80 [mm]	Length of the rubber
$C_{10}$	0.436 [MPa]	Mooney-Rivlin parameter
$C_{01}$	0.109 [MPa]	Mooney-Rivlin parameter
Concave	9 [mm]	Thickness of the concave center

**Table 3:** Parameters of the rubber collector with eccentricity of 4–5 mm.

Name	Value	Note
Rubber_r	36 [mm]	Outer diameter of the rubber
Rubber_thick	16 [mm]	Thickness of the rubber
Rubber_height	105 [mm]	Length of the rubber
$C_{10}$	0.436 [MPa]	Mooney-Rivlin parameter
$C_{01}$	0.109 [MPa]	Mooney-Rivlin parameter
Concave	13 [mm]	Thickness of the concave center

**Table 4:** Statistical information of the collected dataset.

Parameter	Number	Average Value	Variance	Minimum Value	Maximum Value
$K_{L}$	4789	26,594	49,202	0	385,176
$C_{10}$	4789	0.5723	0.3887	0	1.926
$C_{01}$	4789	0.2354	0.2186	0	0.963
Rubber_r	4789	36.3	2.4	30	36
Rubber_thick	4789	14.1	1.99	12	16
Rubber_height	4789	93.2	12.48	80	105
Press	4789	18.78	1.55	0	30
Concave	4789	10.77	2.15	9	14
*K*	4789	7.74 $\times \;10^{6}$	7,475,537	−	2.13$\;\times \;10^{7}$
Bias	4789	3.43	1.55	0	8

**Table 5:** Hyper-parameter settings of the model.

Hyper-Parameter	Value	Note
num_epochs	10	Iterating number of NN model
learning_rate	$1\times 10^{-4}$~$1\times 10^{-2}$	Learning rate of NN model
activation	‘relu’, ‘tanh’, ‘softrelu’	Activation function of NN model
dropout_prob	0–0.5	Dropout of NN model
num_boost_round	100	Iterating number of GBM model
num_leaves	26–66	Leaf number of tree model
num_bag_folds	5	5-fold cross-validation
num_bag_sets	2	Iterating number of Bagging
num_stack_levels	4	Layer number of stacking integration

## Data Availability

The authors do not have permission to share data.
